# Synthetic dural graft septoplasty in epistaxis from hereditary hemorrhagic telangiectasia

**Published:** 2013-09-30

**Authors:** Wilfred Burckhardt B, Claudia Patricia Guerra

**Affiliations:** 1 Universidad del Valle. Hospital Universitario del Valle, Colombia

**Keywords:** hereditary, hemorrhagic telangiectasia, arteriovenous malformation, epistaxis, septoplasty, graft, synthetic dura

## Abstract

It is an autosomal dominant vascular disorder, which has a variety of clinical manifestations, with epistaxis being one of the most common. Many treatment options exist for epistaxis, but with no consensus on which is the method of choice. We describe the case of a patient with hereditary hemorrhagic telangiectasia (HHT) secondary epistaxis with septoplasty managed with synthetic hard graft, which improved intensity and frequency of bleeding episodes. This technique is a variant of the septodermoplasty described by several authors, but the use of synthetic dura can help in obtaining better results and avoid taking skin grafts from other sites different from the surgical site.

## Abstract (es)

Esta condición es un trastorno vascular autosómico dominante, que tiene una variedad de manifestaciones clínicas; entre las más comunes, se encuentra epistaxis. Existen múltiples opciones terapéuticas para la epistaxis, pero no hay un consenso sobre cuál es el método de elección. Se describe el caso de un paciente con epistaxis secundaria a telangiectasias hemorrágicas hereditarias (THH) manejado con septoplastia con injerto de dura sintética, la cual obtuvo mejoría en la intensidad y frecuencia de los episodios de sangrados. Esta técnica es un variante de la septodermoplastia descrita por diversos autores, pero el uso de dura sintética puede ayudar a obtener mejores resultados y evita toma de injertos dérmicos de otros sitios diferentes al sitio quirúrgico.

## Introduction

A great number of topical, systemic, and surgical treatments are available for epistaxis, with variable effectiveness, among them: cauterization, nasal packing, sclerotherapy, embolization, arterial ligation, nasal humidification, laser ablation, septal dermoplasty, and nasal closure; but without consensus on the method of choice. Saunders described this technique for the first time in 1960, consisting in the elimination of the original nasal mucosa and its substitution by a skin graft normally taken from the thigh and sometimes from the oral mucosa. The technique has undergone further modifications, but it generally seems to truly eliminate epistaxis, although later minor interventions are necessary to eliminate peripheral telangiectasias. Diminished sense of smell may be noted, along with increased propensity to develop infections of the sinuses. These adverse effects can influence on the quality of life of patients, given that a secondary effect is that the perception of flavor in foods is affected^1^ ^,^ ^2^.

## Clinical case

**Figure 1:**
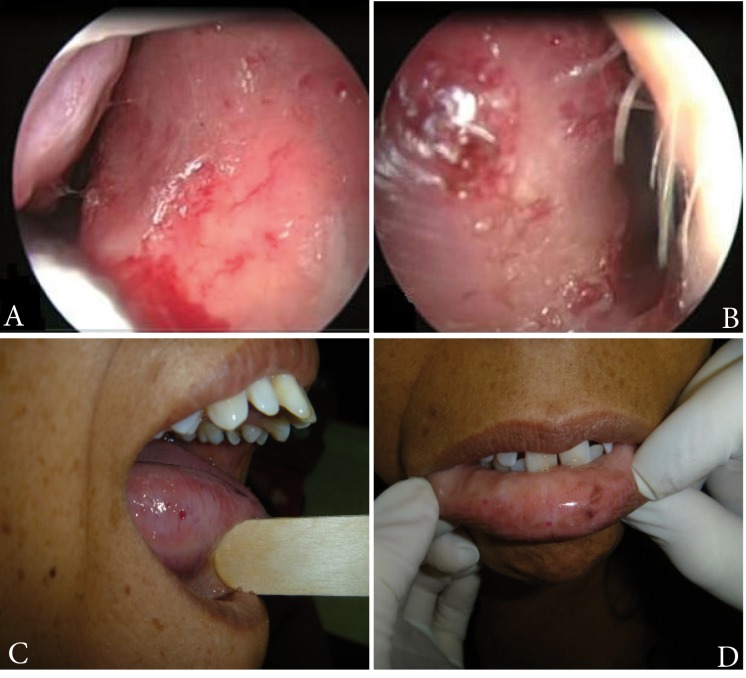
Rhinoscopy with zero-degree endoscopy lens showing telangiectasias on septal mucosa of the right side (A) and left side (B). Telangiectasia on the tongue (C) and lower lip (D).

Patient, 38 years of age, in consultation due to a condition of recurring epistaxis since eight years of age, which appeared with a frequency of two to three episodes per day, in light to moderate amount. On multiple occasions, the patient had required nasal packing and cauterizations. Secondary to the epistaxis, the patient presented iron-deficiency anemia, for which the patient received several blood transfusions. The patient's family group included the patient's mother and a son, both presenting a similar clinical condition. Physical exam evidenced telangiectasias in lower turbinates and bilateral nasal septum, as well as on the oral mucosa and tongue (Figure 1). No cutaneous lesions were found. Upper GI endoscopy was performed, showing a 3-mm ruby-colored flat lesion on the lesser curvature of the stomach (gastric angiodysplasia). Through pulmonary arteriography right pulmonary arteriovenous malformation was detected. 

By applying diagnostic criteria for HHT and complying with all, the definite diagnosis of this condition was considered, without the possibility of genetic tests^1^ ^,^ ^3^.

**Figure 2.**
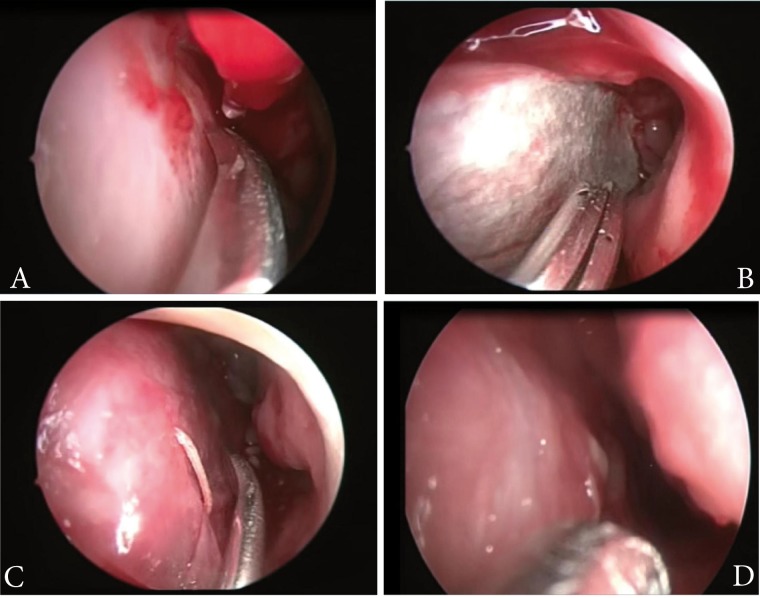
Surgical septoplasty procedure with synthetic dural graft. Left supra-perichondric septal dissection, perichondrium (arrow) and upper mucosa flap (arrow head) (A). Placement of dural graft on the perichondrium, synthetic dura (arrow head) (B) and transfixion suture on anterior septum on dural graft (C). Result after three months of surgery (D)

To control epistaxis episodes, the patient was taken to surgery and a modified septal dermoplasty technique was performed, consisting of a hemitransfixion incision on the caudal septal with No. 15 scalpel blade, followed by a septal dissection at supra-perichondric level with the Cottle dissector, creating an upper and lower ipsilateral tunnel. Then, the septal mucosa was resected leaving a small upper and lower mucosa flap. Synthetic dural graft (Surgisis^(^ Biodesign^TM^ -Dural Graft, REF G31092, made in the United States) was placed on the perichondrium, leaving it covered by the upper and lower mucosa flaps. Thereafter, an absorbable suture stitch was placed transfixing on the anterior and posterior septum; this was then covered with Surgicel^(^. This procedure was carried out unilaterally. During subsequent controls, adequate epithelization of the graft was noted along with decreased episodes of bleeding. After three months, the patient was again taken to surgery to perform this same procedure on the contralateral nostril (Figure 2). After surgery, there was no evidence of septal perforation, infection, telangiectasias on borders, loss of smell, or any other complication, completing one year of postoperative follow up.

## Discussion

Penetrance shows relationship with age and its manifestations increase and develop throughout life. Epistaxis, mucocutaneous telangiectasias, and iron-deficiency anemia stand out among the most important clinical manifestations. Approximately 50% of the individuals diagnosed report epistaxis by 10 years of age and 80-90% by 21 years of age. Up to 95% eventually develop recurrent epistaxis. Epistaxis emerges spontaneously. Many patients with occasional epistaxis episodes only require treatment with iron supplements, while others who experience significant bleeding episodes may require transfusions and diverse forms of nasal interventions^4^.

Other manifestations that can be found in these patients are cutaneous and oral telangiectasias (50-80% of the patients), pulmonary AVM (11-30%), gastrointestinal bleeding (11-40%), hepatic AVM (30%), cerebral AVM (10-15%), and spinal AVM (1%). According to the location of these AVM, clinical findings will emerge like gastrointestinal hemorrhage, dyspnea, thoracic pain, hemothorax, hemoptysis, cyanosis, thoracic breathing, hypoxemia, polycythemia, pulmonary embolism, migraine, convulsions, cerebrovascular events, and transient ischemic accident (11-55%), subarachnoid hemorrhage, cerebral abscess, portal hypertension, among others^2^.

### Confirmation of the diagnosis is obtained through genetic tests for endoglin mutations, ALK-1 or SMAD4 genes.

An important number of topical, systemic, and surgical treatments are available for epistaxis, with variable effectiveness, among which are: cauterization, nasal packing, sclerotherapy, embolization, arterial ligation, nasal humidification, laser ablation, radiofrequency coblation, septal dermoplasty, and nasal closure.^2^ ^,^ ^3^ ^,^ ^5^ ^,^ ^6^

Septal dermoplasty is a skin graft technique. The principle of this procedure is the substitution of the fragile respiratory mucosa of the nose with strong skin grafts (generally from the thigh or oral mucosa) that withstand trauma and, thus, avoid bleeding. Its effectiveness has been objectively demonstrated in reducing the mean number of transfusions required due to epistaxis. The main complications reported with this procedure are: nasal odor (78%), formation of scabs (72%), diminished sense of smell (58%), increased sinus infections (30%). A minority of patients experience worsening of asthma or allergies (8%), facial numbing or tingling (10%), increased eye infections (8%), or nasal pain (14%). Most of the patients, 88%, can breathe through the nose after surgery^7^.

Modification of the septal dermoplasty technique by substituting the skin graft for a synthetic dural graft can diminish the incidence of all these possible complications registered in the literature and also avoids taking skin from a second surgical field, as evidenced in the current case reported where after surgery there was no evidence of septal perforation, infection, telangiectasias on the borders, loss of smell, or any other complication, completing one year of post-operatory follow up.

## Conclusion

An important number of topical, systemic, and surgical treatments are available for epistaxis, with variable effectiveness, but without consensus on the method of choice. Generally, we begin with conservative medical management and if no adequate response is found, the following step involves more invasive interventions. Unlike the septal dermoplasty described in literature, this septoplasty technique with synthetic dural graft avoids taking skin grafts from a second surgical field, with excellent results. After the septal dermoplasty, the literature reports improved quality of life, diminished bleeding episodes, and increased hemoglobin figures. According to this, the patient in the present case has shown clinical improvement, with diminished frequency, amount, and duration time of bleeding. One year after surgery there was no evidence of septal perforation, infection, telangiectasias on the borders, or loss of smell.
